# Impact of free maternity policies in Kenya: an interrupted time-series analysis

**DOI:** 10.1136/bmjgh-2020-003649

**Published:** 2021-06-09

**Authors:** Stacey Orangi, Angela Kairu, Lucas Malla, Joanne Ondera, Boniface Mbuthia, Nirmala Ravishankar, Edwine Barasa

**Affiliations:** 1 Health Economics Research Unit (HERU), KEMRI-Wellcome Trust Research Programme Nairobi, Nairobi, Kenya; 2 Health Services Unit, KEMRI-Wellcome Trust Research Programme Nairobi, Nairobi, Kenya; 3 Independent Consultant, Nairobi, Kenya; 4 ThinkWell, Nairobi, Kenya; 5 ThinkWell, Washington, DC, USA; 6 Centre for Tropical Medicine & Global Health, Nuffield Department of Medicine, University of Oxford, Oxford, UK

**Keywords:** maternal health, health policy, health systems evaluation, health economics

## Abstract

**Background:**

User fees have been reported to limit access to services and increase inequities. As a result, Kenya introduced a free maternity policy in all public facilities in 2013. Subsequently in 2017, the policy was revised to the Linda Mama programme to expand access to private sector, expand the benefit package and change its management.

**Methods:**

An interrupted time-series analysis on facility deliveries, antenatal care (ANC) and postnatal care (PNC) visits data between 2012 and 2019 was used to determine the effect of the two free maternity policies. These data were from 5419 public and 305 private and faith-based facilities across all counties, with data sourced from the health information system. A segmented negative binomial regression with seasonality accounted for, was used to determine the level (immediate) effect and trend (month-on-month) effect of the policies.

**Results:**

The 2013 free-maternity policy led to a 19.6% and 28.9% level increase in normal deliveries and caesarean sections, respectively, in public facilities. There was also a 1.4% trend decrease in caesarean sections in public facilities. A level decrease followed by a trend increase in PNC visits was reported in public facilities. For private and faith-based facilities, there was a level decrease in caesarean sections and ANC visits followed by a trend increase in caeserean sections following the 2013 policy.

Furthermore, the 2017 Linda Mama programme showed a level decrease then a trend increase in PNC visits and a 1.1% trend decrease in caesarean sections in public facilities. In private and faith-based facilities, there was a reported level decrease in normal deliveries and caesarean sections and a trend increase in caesarean sections.

**Conclusion:**

The free maternity policies show mixed effects in increasing access to maternal health services. Emphasis on other accessibility barriers and service delivery challenges alongside user fee removal policies should be addressed to realise maximum benefits in maternal health utilisation.

**Key questions**

**What is already known?**

- Maternal health is still a global challenge especially in sub-Saharan Africa, where there is a lifetime maternal death risk of 1 in 37.
- User fees for financing maternal health is a barrier to access and leads to increased inequities. As a result, Kenya has instituted free maternity policies to increase access to maternal health.

**What are the new findings?**

- This paper estimates the impact of the 2013 free maternity policy and the 2017 Linda Mama programme on the utilisation of the maternal health services in both public and faith-based facilities in Kenya.
- Following the 2013 free maternity policy, there was a level increase in normal deliveries and caesarean sections, then a trend decrease in caesarean sections in public facilities. Additionally, there was a level decrease followed by a trend increase in postnatal care (PNC) visits in public facilities. In the private and faith-based facilities, although a trend decrease in caesarean sections was reported, this was followed by a trend increase. A level decrease in antenatal care (ANC) visits was also reported in private and faith-based facilities.
- After the introduction of the 2017 Linda Mama policy, there was a trend decrease in caesarean sections in the public facilities. There was also a level decrease followed by a trend increase in PNC in public facilities. Private and faith-based facilities had level decrease in normal deliveries and caesarean section, and a trend increase in the latter. No significant effects were reported for ANC and PNC in private and faith-based facilities.

**What do the new findings imply?**

- The free maternity policies’ effects on access to maternal health services are mixed. Attention on other accessibility barriers and service delivery challenges should be considered alongside the financial access barrier.

## Introduction

Maternal health remains a global health challenge in sub-Saharan Africa (SSA) where maternal mortality ratio was estimated at 542 maternal deaths per 100 000 live births in 2017 with a lifetime maternal death risk of 1 in 37.^1^ Skilled care before, during and after delivery can reduce most of these preventable maternal deaths.^2 3^ For example, antenatal care (ANC) ensures good health for the mother and child, and any pregnancy-related complication is detected and appropriately treated; skilled delivery ensures that both complicated and uncomplicated deliveries are proficiently managed; postnatal care (PNC) ensures that severe life-threatening complications that may arise after delivery are detected and well managed.^4–6^ Despite this, Kenya has a 58% coverage of four or more ANC visits, 62% of births are attended by a skilled healthcare provider and 43% of mothers do not receive skilled PNC within the first 6 weeks after delivery.^7^

User fees as a financing mechanism has been reported to be regressive, limits access to services and leads to increased inequities.^8–10^ Specifically, for maternal and child health, a systematic review reported that removal of user fees in low-income and middle-income countries results in an increase in ANC visits and facility deliveries.^11^ Although most of the included studies in the systematic review were of poor quality, more robust evidence done in several countries in SSA confirms that user fee removal is associated with an increase in facility deliveries and a decrease in neonatal mortality.^11 12^ In an effort to increase access, the government of Kenya introduced the free maternity policy in 2013 where all user fees associated with maternal health were removed across all levels of care in the public sector.^13^ Evidence suggests that while this policy was intended to cover ANC, deliveries and PNC, in practice only deliveries were covered.^14^ In 2017, the government of Kenya made a policy decision to move the management of the free maternity programme from the Ministry of Health to the National Hospital Insurance Fund (NHIF) with the focus of improving efficiency, accountability and effectiveness.^15^ The revised free maternity programme labelled ‘Linda Mama’ was introduced in a phased approach with an extension of services beyond the public sector as well as an expansion of the benefit package. Phase 1 of the programme started in April 2017 and involved private-for-profit and faith-based healthcare facilities contracted by the NHIF to offer delivery services. Phase 2 began in July 2017, and all public healthcare facilities were to be contracted to offer delivery services. Finally, phase 3 was rolled out in March 2018 and included expansion of the benefit package to include ANC and PNC care across contracted public, private and faith-based healthcare facilities. Despite the expansion of the benefit package, in practice, the two free maternity policies did not cover the costs of referral.^16^

Previous analysis on the free maternity policy in Kenya has focused on the first free maternity policy.^17–20^ There has been no evaluation of second and current versions of the free maternity policy (Linda Mama) which is distinct from the first in design and implementation arrangements. This paper presents an evaluation of the free maternity policies in Kenya using a quasi-experimental approach. Evaluating the Linda Mama policy will provide evidence to policy-makers about whether (and on what outcomes) the policy is effective and hence inform decisions about whether it is a worthwhile intervention that should be maintained (if effective) or whether there is need to wholly or partly rethink the policy (if found not to be effective).

## Methods

### Study design

We used a retrospective interrupted time-series (ITS) design—which is one of the quasi-experimental designs.^21^ In this design, the data observed before the policies (intervention) would be used as control for the data observed during the intervention period.

### Study setting

The Kenyan government is a devolved government system consisting of the national government and 47 semiautonomous counties.^22^ Health service delivery is devolved and consists of public, for-profit private and the not-for-profit private sector. The latter is mainly faith-based. Healthcare facilities are organised in a hierarchical manner with six levels; community health services (level 1), dispensaries (level 2), health centres (level 3), first referral subcounty hospitals (level 4), second referral county hospitals (level 5), and tertiary referral hospitals (level 6).^23^

**Figure 1:**
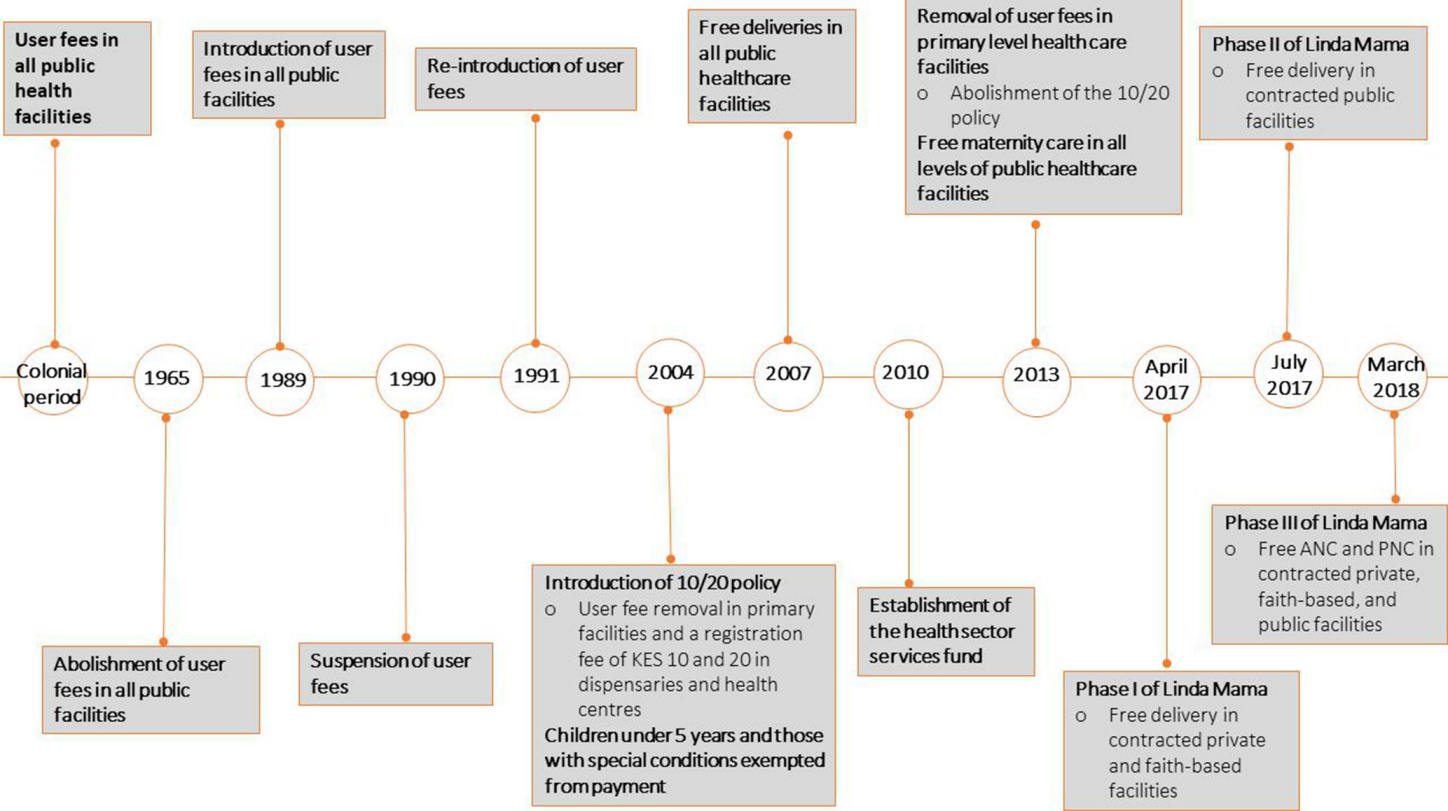
Timeline of user fee reforms in Kenya. ANC, antenatal care; PNC, postnatal care.

In relation to health financing, Kenya has had several user fees reforms over the years, that have had an impact on maternal and child health, illustrated in figure 1. Soon after independence in 1965, user fees were abolished in all public facilities, later in 1989, they were brought back but suspended in 1990 and reintroduced in 1991.^13 24^ Level 4 and 5 public hospitals charged between KES 3000 and 6000 (US$30–60) for caesarean sections, while level 1–5 public facilities would charge patients between KES 700 and 2500 (US$7–25) for a normal delivery. Patients on average were charged KES 150 (US$1.5) for ANC and PNC services in public facilities. In 2004, there was an introduction of the 10/20 policy where user fees were abolished at the primary level and a registration fee of KES 10 and 20 (US$ 0.1 and 0.2) was levied in public dispensaries and health centres, respectively, and services for children under 5 years as well as those with special conditions such as malaria and tuberculosis were exempted from payment.^25^ Later in 2007, there was free deliveries in all public healthcare facilities, however, the extent to which this policy was implemented is unknown.^25^ A health sector services fund was established in 2010 that served to compensate healthcare facilities on lost revenues associated with the user fee removal policies.^13^ Later in June 2013, there was a presidential declaration that led to the abolishment of the 10/20 policy and the introduction of the free maternity policy. Subsequently, the Linda Mama free maternity programme was established in 2017.

This study was conducted across the 47 counties of Kenya and included public, private and faith-based facilities. Specifically, we included 5061 public level 2–3 facilities, 358 public level 4–6 facilities, 210 level 2–3 private and faith-based facilities and 95 level 4–5 private and faith-based facilities. Facilities with 100% missing data across all the utilisation indicators were excluded from the analysis. Private and faith-based facilities were included if listed by the NHIF as offering Linda Mama services.^26^ Five of the counties had universal health coverage (UHC) initiatives in the public sector; four were pilot sites for the country’s UHC programme since December 2018, and the other had a local county run UHC programme, that began in October 2016.

### Data source

The data source for the variables of interest was the Kenya Health Information System which is an open source web-based health information system used for reporting routine data.^27^ The level of missing data for the intervention outcomes was on average, 27% for caesarean sections, 29% for ANC visits, 51% for normal deliveries and 55% for PNC visits. Imputation of the missing data was done at the facility level using structural model and Kalman smoothing approach in instances where data were available for at least 50% of the time points. This imputation approach is recommended for longer and more complex time series that have trend and seasonality, as it very often produces accurate results.^28^

### Study outcomes

We analysed the level and trend changes in intervention outcomes (maternal health utilisation). Specifically, the intervention outcomes were ANC visits, normal deliveries, caesarean sections, and PNC visits.

### Study period

**Figure 2:**
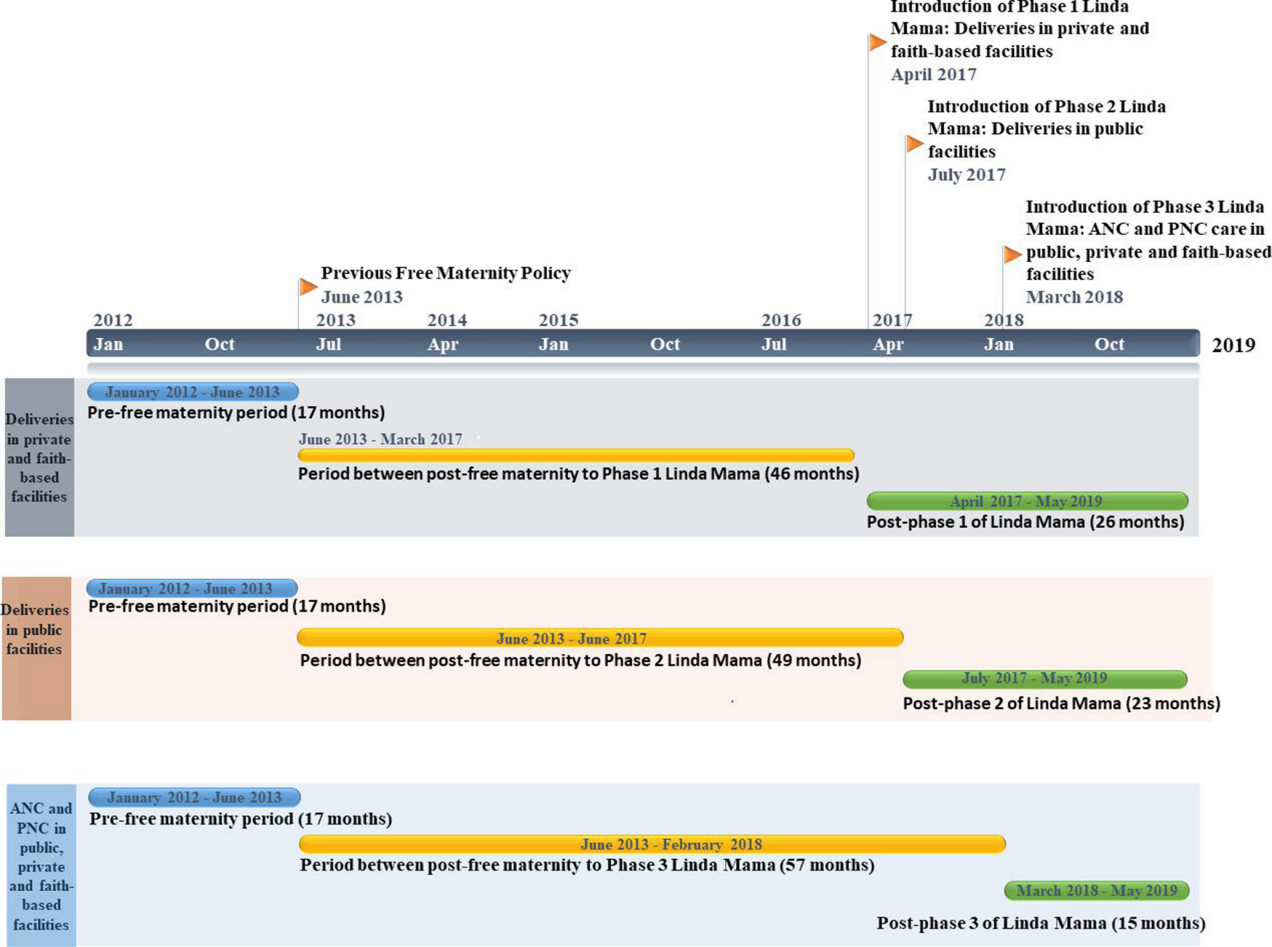
Interrupted time-series study period. ANC, antenatal care; PNC, postnatal care.

The intervention series covered a period of 89 monthly time points from January 2012 to May 2019. The time periods varied based on outcome and facility type due to the phased introduction of the Linda Mama programme that occurred at three different time points as illustrated in figure 2 (ie, phase 1: Introduction of deliveries in faith-based and private facilities in April 2017; phase 2: Introduction of deliveries in public facilities in July 2017 and phase 3: Introduction of ANC and PNC across all types of care in March 2018).

For instance, for deliveries in the private and faith-based facilities, the time periods included 17 months for the prefree maternity policy, 46 months for the period between the onset of the free maternity policy to phase 1 of the Linda Mama policy, and 26 months for the postphase 1 Linda Mama policy period. Deliveries in the public facilities included 17 months for the prefree maternity policy, 49 months for the period between the onset of the previous free maternity policy to phase 2 of the Linda Mama policy and 23 months for the postphase 2 Linda Mama policy period. ANC and PNC in public, private and faith-based facilities included 17 months for the prefree maternity policy period, 57 months for the period between the onset of the previous free maternity policy and phase 3 of the Linda Mama policy, and 15 months for the period postphase 3 of the Linda Mama policy. A total wash out period of 16 months was included, at different time points, that captured eight nationwide health workers strikes from 2012 to 2017.^29 30^ These washout periods were March 2012; September 2012–October 2012; December 2012–February 2013; December 2013; December 2016–March 2017 and June 2017–October 2017.

Two interruptions were placed for each intervention outcome. The first interruption represented the introduction of the original free maternity policy and the second interruption represented one of the three phases of the Linda Mama programme introduction; June 2013 and April 2017 for deliveries in private and faith-based facilities; June 2013 and July 2017 for deliveries in public facilities and June 2013 and March 2018 for ANC and PNC services in private, faith-based and public facilities.

### Statistical analysis

#### Primary analysis

We estimated the level and trend changes in the intervention outcomes. The analysis included the following independent variables; time (T) which was coded sequentially from 1 to 89; intervention status (X) which was defined as 0 for prefree maternity period, 1 for the period between the onset of free maternity policy and before Linda Mama, 2 for the periods were there were nationwide healthcare workers strikes and 3 for the period after the onset of the Linda Mama policy. The health workers strike period was used as a wash out period proxy. This is because during this time, utilisation of maternal health services was disrupted and does not reflect the true effect of the free maternity policies.

The outcome variables were plotted against time to visually inspect the data for outliers, trends and seasonality. Initial analyses suggested overdispersion of the outcome variables, therefore a negative binomial distribution was assumed for the outcomes. Three different models were fitted for each of the four intervention outcome variables, one that had all 89 time points and did not define the wash out period, another that defined the wash out period and the last that excluded the wash out period. Adjustments were made for any seasonal effects by using harmonic terms based on the month of the year.^31^ We used the Akaike Information Criteria (AIC) to determine the best fitting model. The final model was expressed as follows:

$$Y_{t}=B_{0}+B_{1}T_{t}+B_{2}X_{t}+B_{3}X_{t}T_{t}$$

Where B_0_ is the baseline level at time 0, B_1_ represents the *trend* change in the preintervention phase, B_2_ represents the level change following the intervention and B_3_ represents the *trend* change following the intervention.

#### Secondary analyses

We considered *four* forms of secondary analyses. The first assessed whether the observed level and trend changes were attributable to the implemented policies. This involved the use of non-intervention outcome as a control. Out-patient day (OPD) visits was included to control for ANC and PNC visits, while inpatient admissions in public facilities and faith-based facilities was chosen as a control for normal and caesarean sections. The free maternity policies were aimed at maternal health and therefore the controls were not directly targeted by the policies. An additional variable (Z) was added that denotes the type of outcome (whether treatment or control), and as a result, we fitted a controlled interrupted time-series (CITS) model of the form:

$$Y_{t}=B_{0}+B_{1}T_{t}+B_{2}X_{t}+B_{3}X_{t}T_{t}+B_{4}Z+B_{5}ZT_{t}+B_{6}ZX_{t}+B_{7}ZX_{t}T_{t}$$

Where B_4_ is the difference in intercept at time 0, B_5_ defines the trend difference between the intervention and control group in the preintervention period, B_6_ defines difference between the change in level in the control and intervention group associated with the intervention and finally B_7_ is the difference between the trend change in the control and intervention group associated with the intervention. We interpret B_6_ and B_7_ to infer any causal effects.

In the second form of the secondary analysis, we conducted a combined ITS comparing the difference between the level and trend change in the five counties that had UHC initiatives in the public sector and the remaining counties that did not have any UHC initiatives.

Third, we did an available case analysis where a separate ITS for the intervention outcomes without imputing for any missing data was done.

Finally, the fourth secondary analysis involved the use of pseudo-start periods (replacing the true intervention start dates with other start dates along the preintervention period) for the intervention outcomes which had a pre-existing trend prior to the intervention.

#### Model diagnostics

The AICs of the different regression models for both the separate intervention outcome models and the single CITS are shown in online supplemental table 1. For all the outcomes, the best-fitting model excluded the health worker strike periods, with or without accounting for seasonal trends.

10.1136/bmjgh-2020-003649.supp1 Supplementary data

**Figure 3:**
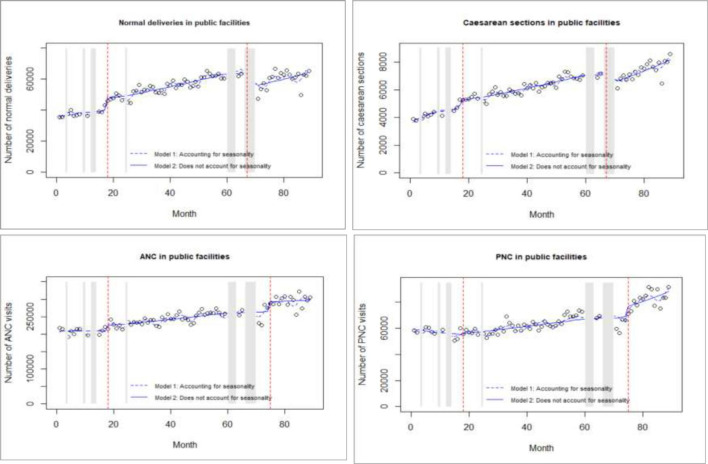
Interrupted time-series analysis for intervention outcomes in public facilities. ANC, antenatal care; PNC, postnatal care.

**Figure 4:**
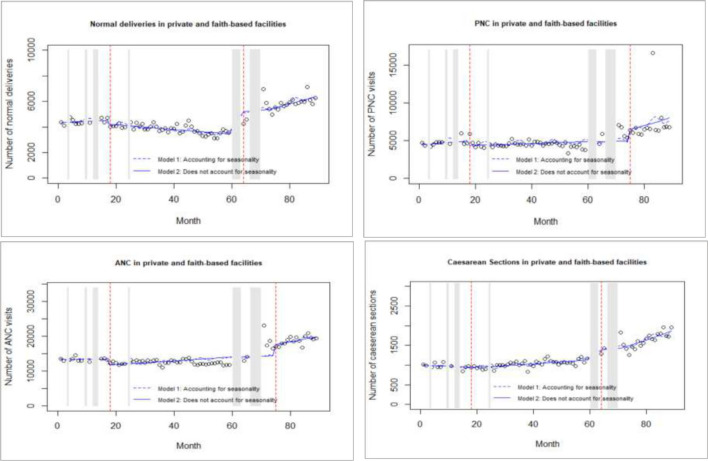
Interrupted time-series analysis for intervention outcomes in private and faith-based facilities. ANC, antenatal care; PNC, postnatal care.

The model diagnostics included examination of residuals, autocorrelation function as well as partial autocorrelation function. table 1 reports the best-fitted negative binomial model estimates for the separate ITS for the intervention outcomes in public, private and faith-based facilities, while figures 3 and 4 visualise predicted numbers from the model that account and do not account for seasonality. The final model estimates for the control outcomes in the separate ITS analysis are reported in online supplemental table 2, while online supplemental table 3 reports on the model estimates for the single CITS.

10.1136/bmjgh-2020-003649.supp2 Supplementary data

10.1136/bmjgh-2020-003649.supp3 Supplementary data

**Table 1:** Final negative binomial estimates for the intervention outcomes in the separate ITS analysis

	Public facilities							
	Normal delivery		Caesarean section		ANC		PNC	
	Estimate (95% CI)	P value	Estimate (95% CI)	P value	Estimate (95% CI)	P value	Estimate (95% CI)	P value
Slope change prepolicy	1.005 (0.997 to 1.014)	0.234	**1.022 (1.013 to 1.030**)	**0.000**	1.001 (0.992 to 1.009)	0.899	**0.989 (0.979 to 0.999**)	**0.026**
Effect of free maternity policy								
Level change	**1.196 (1.113 to 1.286**)	**0.000**	**1.289 (1.207 to 1.377**)	**0.000**	1.031 (0.966 to 1.101)	0.359	**0.856 (0.792 to 0.926**)	**0.000**
Trend change	1.002 (0.993 to 1.011)	0.725	**0.986 (0.978 to 0.994**)	**0.001**	1.003 (0.995 to 1.012)	0.415	**1.016 (1.006 to 1.026**)	**0.002**
Effect of Linda Mama policy								
Level change	1.129 (0.876 to 1.456)	0.349	1.000 (0.795 to 1.256	0.997	1.223 (0.871 to 1.718)	0.246	**0.619 (0.412 to 0.929**)	**0.021**
Trend change	1.001 (0.991 to 1.010)	0.903	**0.989 (0.980 to 0.998**)	**0.016**	1.002 (0.992 to 1.012)	0.714	**1.023 (1.011 to 1.035**)	**0.000**
Private and faith-based facilities								
Slope change prepolicy	1.003 (0.990 to 1.016)	0.667	0.992 (0.980 to 1.004)	0.196	1.002 (0.983 to 1.022)	0.803	1.011 (0.981 to 1.043)	0.477
Effect of free maternity policy								
Level change	1.020 (0.916 to 1.137)	0.717	**0.854 (0.773 to 0.944**)	**0.002**	**0.850 (0.729 to 0.991**)	**0.038**	0.962 (0.753 to 1.229)	0.755
Trend change	0.992 (0.979 to 1.005)	0.233	**1.013 (1.000 to 1.025)**	**0.044**	1.002 (0.983 to 1.021)	0.855	0.992 (0.961 to 1.023)	0.591
Effect of Linda Mama policy								
Level change	**0.681 (0.494 to 0.938**)	**0.019**	**0.603 (0.454 to 0.801**)	**0.000**	0.831 (0.374 to 1.848)	0.65	0.620 (0.171 to 2.244)	0.466
Trend change	1.008 (0.993 to 1.022)	0.298	**1.024 (1.010 to 1.037**)	**0.000**	1.006 (0.983 to 1.029)	0.626	1.004 (0.968 to 1.041)	0.847

All segmented regression used a log link function with negative binomial distribution and p values are derived from z tests. Values in bold represent a strong evidence of an effect at a 0.05 level of significance.

ANC, antenatal care; ITS, interrupted time series; PNC, postnatal care.

In all the fitted and interpreted models in the primary analysis, the residual (see online supplemental figure 1), partial autocorrelation and autocorrelation (online supplemental figures 2 and 3) plots showed no evidence of autocorrelation in the data.

10.1136/bmjgh-2020-003649.supp4 Supplementary data

10.1136/bmjgh-2020-003649.supp5 Supplementary data

10.1136/bmjgh-2020-003649.supp6 Supplementary data

### Ethics approval

Ethics approval to conduct the study was obtained from the Kenya Medical Research Institute/Scientific and Ethics Review Unit (KEMRI/SERU/CGMR-C/132/3735). We also obtained approvals from the Council of Governors, National Commission for Science, Technology and Innovation, the respective county department of health, and the health facilities management.

### Patient and public involvement

No patients were involved in this ITS analysis.

## Results

A total number of 4 493 491 normal deliveries, 510 261 caesarean sections, 21 021 591 ANC visits and 5 570 609 PNC visits from public facilities were included in the primary ITS analysis. For private and faith-based facilities, a total of 473 167 normal deliveries, 121 616 caesarean sections, 1 348 834 ANC visits and 495 925 PNC visits were included in the ITS.

### Effect of the 2013 free maternity policy

#### Effect in public facilities

The 2013 free maternity policy resulted in a significant 19.6% and 28.9% level increase in normal deliveries and caesarean sections in public facilities, respectively. The latter was followed by a 1.4% month-on-month trend decrease in caesarean sections. The policy also resulted in a 14.4% level decrease in PNC visits followed by a 1.6% month-on-month trend increase in public facilities. Conversely, there was no level or trend effects of the policy on ANC visits.

The results also suggest that prior to the 2013 policy, there was an existing trend increase in caesarean sections and a trend decrease in PNC visits.

#### Effect in private and faith-based facilities

In private and faith-based facilities, there was a reported 14.6% level decrease in caesarean sections followed by a 1.3% trend increase in the same service as a result of the 2013 free maternity policy. There was also a significant 15% level decrease in ANC following the policy. Despite the above, there was no statistically significant effect in the level or trend of PNC visits and normal deliveries in private and faith-based facilities.

### Effect of the 2017 Linda Mama policy

#### Effect in public facilities

The introduction of the Linda mama policy in 2017 did not have a significant effect on the level and trend of utilisation of normal deliveries and ANC visits in public facilities. The policy however led to a 1.1% month-on-month trend decrease in caesarean sections. Additionally, a 38.1% decrease in PNC visits followed by a 2.3% month-on-month increase in PNC visits was reported in public facilities.

#### Effect in private and faith-based facilities

The Linda Mama policy had no statistically significant effect on either the level or trend of utilisation of ANC and PNC in private and faith-based facilities. However, there was a 31.9% level decrease in normal deliveries and a 39.7% decrease in caesarean sections in faith-based facilities following the Linda Mama policy. A trend increase (2.4%) in caesarean sections was also reported.

### Secondary analyses

After accounting for confounding, the CITS analysis produced similar results in several cases. Specifically, the direction of effect observed in the primary analysis was similarly reported in the differential level and trend changes in the CITS. The significant magnitude of effects changed slightly in the CITS as compared with the primary analysis. On the other hand, there were instances where, the reported significant effects in the primary analysis differed from the CITS. These should be interpreted with caution as they may be indicative of other cointerventions. Finally, in the CITS, there was also some significant differential level and trend changes that were not observed in the separate ITS, which could be suggestive of a change in the control outcome independent of the intervention. The CITS is illustrated in online supplemental table 3.

The second secondary analysis reported no significant additional change in level and trend effects of maternal health utilisation outcomes in counties with UHC initiatives over and above those that did not have UHC initiatives. This is illustrated in online supplemental table 4.

10.1136/bmjgh-2020-003649.supp7 Supplementary data

The secondary analysis where no imputation was done (available case analysis) to a large extent had similar significant level and trend effects to the primary analysis especially in terms of the direction of effect. This is except for some effects on PNC in public facilities, and ANC and caesarean sections in private and faith-based facilities. Conversely, effects in ANC visits in public facilities, and normal and PNC visits in private and faith-based facilities were observed in the secondary analysis but not in the primary analysis. The results of this secondary analysis are illustrated in online supplemental table 5.

10.1136/bmjgh-2020-003649.supp8 Supplementary data

Second, a pseudostart intervention period, 3 months prior to the 2013 free maternity policy was determined not to elicit a pre-existing trend for normal deliveries, caesarean sections and PNC visits in public facilities.

## Discussion

This study evaluates the effects of both the previous free maternity policy and the revised Linda Mama free maternity policy in increasing the utilisation of essential maternal health services in Kenya. This was achieved by using an ITS analysis which is a powerful quasiexperimental design used to evaluate the effectiveness of population level health interventions.^32^

Our study detected a significant 19.6% and 28.9% level increase in the number of normal deliveries and caesarean sections, respectively, in public facilities after the introduction of the previous free maternity policy in 2013. There was no significant trend effect in normal deliveries, but there was a 1.4% month-on-month trend decrease in caesarean sections following this same policy. These findings differ with prior studies that showed an increase in trend of health facility deliveries following the same policy.^17 18^ Nonetheless, our findings are similar to a study that reports a short-term increase in the number of caesarean sections in one county referral hospital following the previous free maternity policy, however this later diminished in significance with time.^19^

Our findings on the lack of sustained trend effects in normal deliveries and the decrease in trend in caesarean sections over time in public facilities following the policy, could be as a result of two factors. First, evidence suggests that the implementation of the 2013 free maternity policy was not matched by supply side strengthening.^14 33^ As a result, the lack of adequate drugs, equipment, infrastructure and skilled human resources may have inhibited facilities from conducting normal deliveries and caesarean sections when needed but rather led to referrals to other facilities. Second, the provider payment mechanisms adapted in the free maternity programme were similar case-based rates to reimburse healthcare facilities regardless of the type of delivery.^14^ For instance, county and subcounty hospitals were reimbursed KES 5000 (US$ 50), while health centres and dispensaries were reimbursed KES 2500 (US$25) for each delivery regardless of whether it was a normal delivery or caesarean section. Tertiary referral hospitals were reimbursed KES 17 000 (US$170) regardless of the type of delivery due to the higher likelihood of them handling complicated cases.^14^ Conversely, the NHIF pays these same healthcare facilities KES 10 000 (US$100) for a normal delivery and KES 30 000 (US$300) for a caesarean section for beneficiaries enrolled in the NHIF national scheme. These payment rates for the free maternity programme have been reported to be inadequate, leading to a strain in resources.^16^ This may have disincentivised providers from conducting normal deliveries and elective caesarean sections under the free maternity programme, due to the inadequacy of the reimbursements to cover incurred costs and rather opting to refer them. Indeed, when we compared the proportion of caesarean sections in the Linda Mama programme to that in the NHIF national scheme, we found that for the financial year 2019, the proportion caesarean sections in the Linda Mama programme was 8%, while that in the NHIF general scheme was 34% (online supplemental figure 4). This high proportion of caesarean section in the national scheme may also be because of perverse incentives for providers to conduct unnecessary caesarean sections for financial reasons. This is illustrative of the need to carefully calibrate and align provider payment rates across the different NHIF schemes to ensure they adequately reimburse facilities, do not result in perverse incentives, and accompany them with effective monitoring processes.

10.1136/bmjgh-2020-003649.supp9 Supplementary data

In the private and faith-based facilities, there was no effect on normal deliveries, but there was a level decrease in caesarean sections following the previous free maternity policy. This is expected due to the restriction of the previous free maternity policy to the public sector.

The 2013 previous free maternity policy had no level and trend effects on ANC visits in public healthcare facilities. There was, however, a level decrease in PNC visits in the public facilities, followed by a month-on-month trend increase in the same in public facilities following this policy. The lack of significant level effects on ANC visits and the reduced level effects in PNC visits were probably due to the lack of clarity on the inclusion of ANC and PNC in the benefit package at the onset of the previous free maternity policy.^14^ Evidence suggests that later on into the programme, there was communication for their inclusion, but this was not always adhered to.^14^ An increase in ANC attendance in public healthcare facilities in Kenya after the introduction of the previous free maternity policy has however been reported in other studies.^18 34^ In the private and faith-based facilities, there were no significant level or trend effects on PNC visits and normal deliveries, but there was a level decrease in ANC visits and caesarean sections. This could be as a result of the restriction of the previous free maternity policy to the public sector.

Literature suggests that costs are a barrier to access of maternal health services in Kenya with majority of the poor delivering at home.^35 36^ User fee removal for maternal health has been shown to increase health facility delivery among the poor in Kenya.^37^ Similar trends have been observed in Burkina Faso, Mali, Benin and Malawi where quasiexperimental designs showed an increase in facility-based deliveries following user fee removal policies.^38–40^ Additionally, Malawi reported an increase in ANC visits following the user fee removal in mission facilities.^40^

After the revision of the free maternity policy into the Linda Mama programme almost 4 years later, our results suggest that there were no significant effects on the level and trend of normal deliveries in public facilities. However, there was a trend decrease in caesarean sections in public facilities over time. This decrease in trend effects in caesarean sections and a lack of sustained trend effects in normal deliveries implies that the Linda Mama programme did not further reduce any financial barriers in addition to what the previous free maternity policy afforded.

The revision of the policy came with an introduction of case-based payment rates for ANC and PNC that were not included in the previous programme. Despite this, there was no reported effect on ANC utilisation in the public facilities. However, there was a significant level decrease in PNC followed by a trend increase in the same in public facilities. The latter trend increase was as a result of PNC being included in the revised benefit package. The lack of sustained trend effects in most maternal health service utilisation outcomes in the public facilities could also be as a result of the persisting implementation fidelity challenges of the programme.^16^ For instance, it has been reported that despite the expanded benefit package under the Linda Mama programme, there was inadequate communication and a lack of clarity on the benefit package among both the implementers and beneficiaries.^16^ Additionally in some cases, out of pocket payments were still being levied to beneficiaries of the Linda Mama programme.^16^ While we expected to see an increase in maternal health utilisation in the private and faith-based facilities because the programme increased access beyond the public sector, our findings report no level or trend effects observed in ANC and PNC visits. Additionally, there was no trend effects in normal deliveries, but a significant level decrease in both normal and caesarean sections was reported. This is perhaps because the provider payment mechanisms covered only a small portion of the costs incurred in private sector to offer these services: evidence suggests that the Linda Mama reimbursement rates were deemed insufficient to cover the costs incurred and additionally there were delays and an unpredictability in timings and amount reimbursed, which lead to out of pocket payments in some cases.^16^

The use of longitudinal data in conducting the time-series analysis of the two policies was useful in showing both the immediate and sustained effects however, we ran the risk of history bias.^21 41^ A secondary analysis using a single controlled interaction model to account for possible confounders was done. However, the free maternity policies were rolled out nationally and implementation was in all public health facilities in Kenya. This provided limitations in getting controls in health facilities where the policy was not implemented for comparison. As a result, our choice of non-intervention outcomes was limited to characteristic-based controls (controls chosen from groups with characteristics different to those targeted by the policies). In which case, population groups other than those coming to receive maternal healthcare in the facilities were considered. Vertical programmes such as HIV tests were deemed not appropriate as controls as they may not be exposed to similar cointerventions and confounders. As a result, OPD visits and inpatient admissions in public hospitals, private and faith-based facilities were selected as they were not targeted during the implementation of the policies but could be exposed to similar confounders as maternal health. However, if the free maternity policies had an indirect effect on utilisation of other services other than maternal health, this effect would not be disentangled in our analysis, presenting a limitation in the use of controls for this analysis.

This study presents other notable limitations. First, we used data from the Kenya Health Information System which may not always conform to the information reported in facility records. Additionally, the utilisation variables from this data source had about 27%–55% missing data. Although imputation was done, this could have influenced our observed results. Second, private and faith-based facilities are contracted by NHIF to offer Linda Mama services. Therefore, the start period for offering these services could differ widely and, in some cases, the facilities may opt not to continue offering maternal services under the Linda Mama programme. The above have not been captured in our analysis and an assumption is made that all these facilities offered Linda Mama services from the start of the policy and continue to do so for the time period of the analysis. Third, this analysis did not consider other barriers to access and we recommend further analysis on this. Despite this, our study fills an evidence gap in providing useful insights on the effect of the free maternity policies in Kenya.

In conclusion, our study shows that the effect of user fee removal policies in increasing access to maternal health services has been mixed. For instance, while the public facilities reported a level increase in normal deliveries, and caesarean sections in 2013 and a trend increase in PNC in 2013 and 2017, there have been either no sustained trend effects or a decrease in trend effects in most maternal health services in public and private facilities. This emphasises the importance of addressing service delivery issues such as supply-side strengthening, effective communication and clarity of policy, and implementation fidelity alongside removing the financial access barrier. Additionally, these policies represent opportunities for enhancing strategic purchasing for high priority maternal and neonatal health services. Finally, an increase in utilisation of maternal health services may also call for multisectorial approaches to ensure that geographic accessibility is improved on.

## Data Availability

Data are available upon request. The data set and statistical code is available from the corresponding author (SO) upon reasonable request.
