# Supplementary material for: Impact of free maternity policies in Kenya: an interrupted time-series analysis
Source: BMJ Glob Health. 2021 Jun 9;6(6):e003649. doi: 10.1136/bmjgh-2020-003649 (PMC8191610; doi:10.1136/bmjgh-2020-003649)
Supplement: Supplementary data [file bmjgh-2020-003649supp001.pdf]

Supplementary Table 1: AIC of the different negative binomial models for both the separate intervention and control ITS and the single CITS

Separate Intervention ITS Models									Separate Control ITS Models			
	**Normal deliveries**		**Caesarean section**		**ANC**		**PNC**		**Outpatient**		**Admissions**	
	**Public facilities**	**Private &FB facilities**	**Public facilities**	**Private &FB facilities**	**Public facilities**	**Private &FB facilities**	**Public facilities**	**Private &FB facilities**	**Public facilities**	**Private &FB facilities**	**Public facilities**	**Private &FB facilities**
Model 1: Does not define wash out period	1854.78	1552.42	1497.60	1286.20	2140.17	1669.23	1867.95	1530.13	2397.17	2078.58	2077.40	1648.056
Model2: Does not define wash out period and adjusted for seasonality	1848.16	1553.02	1495.10	1286.90	2141.53	1667.39	1871.40	1531.80	2393.65	2075.60	2081.83	1650.20
Model 3: Defines wash out period	1744.97	1389.19	1371.26	1115.82	2086.29	1624.31	1818.66	1479.62	2336.18	2044.86	1980.54	1574.19
Model 4: Defines wash out period and adjusted for seasonality	1731.71	1387.77	1365.11	1113.17	2078.74	1626.99	1822.24	1481.79	2323.20	2045.60	1977.65	1572.84
Model 5: Excludes wash out period	1392.28	1066.55	1047.03	858.59	1598.42	**1281.41**	1427.33	**1202.31**	1876.35	**1554.64**	1525.90	1268.93
Model 6: Excludes wash out period and accounts for seasonality	**1369.56**	**1062.95**	**1038.06**	**853.7**	**1581.73**	1287.65	**1412.17**	1203.33	**1848.47**	1556.41	**1508.81**	**1261.22**

Controlled Interrupted Time Series Models										
	**Normal deliveries**		**Caesarean section**		**ANC**		**PNC**			
	**Public facilities**	**Private &FB facilities**	**Public facilities**	**Private &FB facilities**	**Public facilities**	**Private &FB facilities**	**Public facilities**	**Private &FB facilities**		
Model 1: Does not define wash out period	3993.71	3228.98	3615.56	2949.99	4530.711	3760.09	4268.70	3605.47		
Model 2: Does not define wash out period and adjusted for seasonality	3993.25	3227.21	3615.25	2947.85	4523.52	3760.60	4267.39	3603.25		
Model 3: Defines washout period	3797.31	2962.29	3413.04	2689.23	4421.83	3694.65	4160.82	3534.48		
Model4: Defines wash out period and adjusted for seasonality	3789.52	2963.52	3409.98	2687.39	4399.65	3697.89	4154.59	3534.04		
Model 5: Excludes wash out period	2930.40	**2335.92**	2600.47	**2133.09**	3498.29	**2834.48**	3315.43	**2766.53**		
Model 6: Excludes wash out period and accounts for seasonality	**2901.41**	2342.09	**2579.42**	2134.93	**3452.68**	2836.98	**3298.43**	2770.32		

Values in bold represent the best fitting models
