# Supplementary material for: Impact of free maternity policies in Kenya: an interrupted time-series analysis
Source: BMJ Glob Health. 2021 Jun 9;6(6):e003649. doi: 10.1136/bmjgh-2020-003649 (PMC8191610; doi:10.1136/bmjgh-2020-003649)
Supplement: Supplementary data [file bmjgh-2020-003649supp002.pdf]

Supplementary Table 2: Final negative binomial estimates for the control outcomes in the separate ITS analysis

	Public facilities			
	OPD visits		Inpatient Admissions	
	Estimate (95% CI)	p-value	Estimate (95% CI)	p-value
**Slope change pre-policy**	1.000(0.986-1.014)	0.958	0.989(0.975-1.003)	0.130
**Effect of free maternity policy**				
Level change	1.102(0.986-1.232)	0.086	1.113(0.991-1.251)	0.072
Trend change	1.003(0.989-1.017)	0.684	1.010(0.995-1.025)	0.179
**Effect of Linda Mama policy**				
Level change	0.687(0.385-1.225)	0.203	**0.415(0.275-0.628)**	**0.000**
Trend change	1.011(0.995-1.028)	0.187	**1.026(1.010-1.042)**	**0.002**
	**Private and faith-based facilities**			
**Slope change pre-policy**	1.010(0.990-1.031)	0.320	**1.017(1.001-1.032)**	**0.036**
**Effect of free maternity policy**				
Level change	0.859(0.730-1.012)	0.069	**1.321(1.167-1.497)**	**0.000**
Trend change	1.004(0.984-1.025)	0.680	**0.981(0.966-0.996)**	**0.015**
**Effect of Linda Mama policy**				
Level change	1.534 (0.651-3.614)	0.328	0.900(0.621-1.305)	0.579
Trend change	0.999(0.975-1.023)	0.912	0.991(0.975-1.00)7	0.271

All segmented regression used a log link function with negative binomial distribution and p-values are derived from z-tests. Values in bold represent a strong evidence of an effect at a 0.05 level of significance
