# Supplementary material for: Impact of free maternity policies in Kenya: an interrupted time-series analysis
Source: BMJ Glob Health. 2021 Jun 9;6(6):e003649. doi: 10.1136/bmjgh-2020-003649 (PMC8191610; doi:10.1136/bmjgh-2020-003649)
Supplement: Supplementary data [file bmjgh-2020-003649supp003.pdf]

Supplementary Table 3: Final negative binomial estimates for the single controlled interrupted time series in public and faith-based facilities

**Maternal utilization versus Control (OPD visits/Inpatient admissions)**	**Public facilities**							
	**Normal delivery**		**Caesarean section**		**ANC**		**PNC**	
	Estimate (95% CI)	p-value	Estimate (95% CI)	p-value	Estimate (95% CI)	p-value	Estimate (95% CI)	p-value
**Slope change pre-policy**								
Diff between intervention and control	**1.022(1.004-1.040)**	**0.014**	**1.036(1.019-1.055)**	**0.000**	1.001(0.985-1.018)	0.889	0.995(0.976-1.014)	0.583
**Effect of free maternity policy**								
Differential level change	1.115 (0.996-1.286)	0.136	**1.186(1.030-1.367)**	**0.018**	0.945(0.829-1.078)	0.403	**0.820(0.705-0.954)**	**0.010**
Differential trend change	0.987 (0.969-1.004)	0.139	**0.973(0.956-0.990)**	**0.002**	1.000(0.984-1.017)	0.958	1.008(0.989-1.027)	0.425
**Effect of Linda Mama policy**								
Differential level change	**2.731(1.643-4.538)**	**0.000**	**2.541(1.540-4.191)**	**0.000**	1.660(0.833-3.309)	0.150	0.916(0.414-2.028)	0.829
Differential trend change	**0.971(0.953-0.990)**	**0.003**	**0.961(0.943-0.980)**	**0.000**	0.992(0.973-1.011)	0.401	1.007(0.985-1.030)	0.547
	**Private and faith-based facilities**							
**Slope change pre-policy**								
Diff between intervention and control	0.989(0.968-1.011)	0.330	**0.979(0.958-1.000)**	**0.047**	0.989(0.962-1.017)	0.449	1.001(0.965-1.038)	0.966
**Effect of free maternity policy**								
Differential level change	**0.793(0.663-0.949)**	**0.011**	**0.667(0.559-0.796)**	**0.000**	0.973(0.778-1.215)	0.805	1.119(0.834-1.502)	0.454
Differential trend change	1.009(0.987-1.031)	0.423	**1.030(1.008-1.052)**	**0.007**	1.000(0.973-1.029)	0.975	0.987(0.951-1.025)	0.498
**Effect of Linda Mama policy**								
Differential level change	0.789(0.461-1.351)	0.388	0.709(0.420-1.198)	0.199	0.511(0.159-1.646)	0.261	0.404(0.086-1.893)	0.250
Differential trend change	1.014(0.991-1.038)	0.239	**1.030(1.006-1.053)**	**0.012**	1.011(0.978-1.044)	0.530	1.005(0.962-1.050)	0.824

All segmented regression used a log link-function with negative binomial distribution and p-values are derived from z-tests. Values in bold represent significant effects at a 0.05 level of significance
