# Supplementary figures and images for: Impact of free maternity policies in Kenya: an interrupted time-series analysis
Source: BMJ Glob Health. 2021 Jun 9;6(6):e003649. doi: 10.1136/bmjgh-2020-003649 (PMC8191610; doi:10.1136/bmjgh-2020-003649)

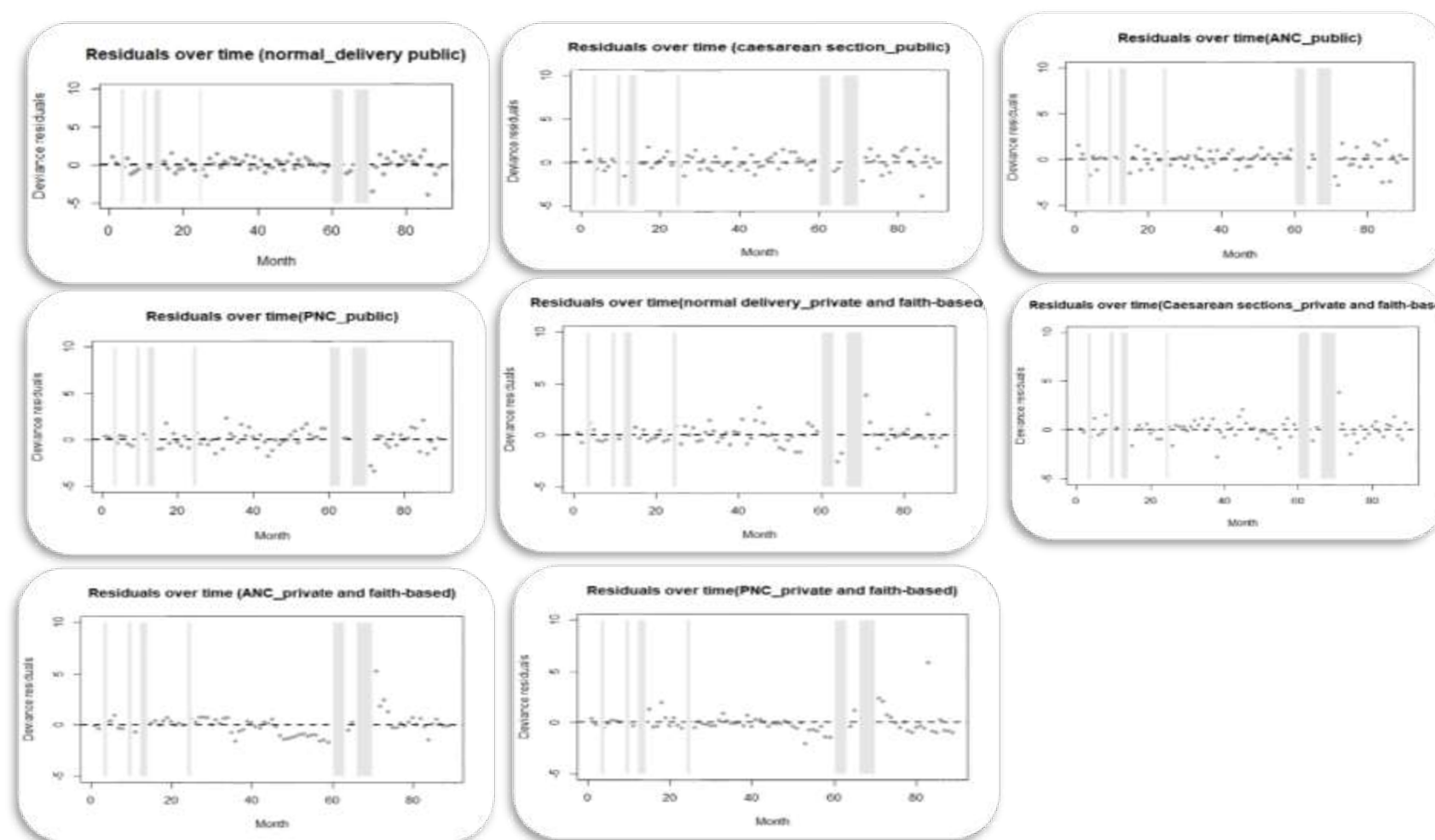

Supplementary Figure 1: Residual plots for the separate ITS for the intervention outcomes

Supplement: Supplementary data [file bmjgh-2020-003649supp004.pdf]
