# Supplementary material for: Impact of free maternity policies in Kenya: an interrupted time-series analysis
Source: BMJ Glob Health. 2021 Jun 9;6(6):e003649. doi: 10.1136/bmjgh-2020-003649 (PMC8191610; doi:10.1136/bmjgh-2020-003649)
Supplement: Supplementary data [file bmjgh-2020-003649supp007.pdf]

Supplementary table 4: Final negative binomial estimates for the combined interrupted time series comparing counties with and without UHC initiatives in public facilities

**Counties with UHC initiatives versus those with no UHC initiative**	**Public facilities**							
	**Normal delivery**		**Caesarean section**		**ANC**		**PNC**	
	Estimate (95% CI)	p-value	Estimate (95% CI)	p-value	Estimate (95% CI)	p-value	Estimate (95% CI)	p-value
**Effect of free maternity policy**								
Differential level change	0.903(0.802-1.016)	0.091	0.916(0.807-1.040)	0.177	0.984(0.896-1.079)	0.727	1.028(0.869-1.216)	0.748
Differential trend change	0.999(0.985-1.014)	0.913	1.008(0.992-1.024)	0.334	0.998(0.987-1.010)	0.770	0.994(0.973-1.015)	0.552
**Effect of Linda Mama policy**								
Differential level change	0.919(0.605-1.395)	0.691	0.907(0.593-1.390)	0.655	1.018(0.625-1.658)	0.943	1.249(0.518-3.012)	0.620
Differential trend change	0.999(0.984-1.015)	0.950	1.008(0.991-1.025)	0.358	0.998(0.984-1.012)	0.775	0.990(0.966-1.015)	0.415

All segmented regression used a log link function and p-values are derived from z-tests. Values in bold represent significant effects at a 0.05 level of significance
