# Supplementary material for: Impact of free maternity policies in Kenya: an interrupted time-series analysis
Source: BMJ Glob Health. 2021 Jun 9;6(6):e003649. doi: 10.1136/bmjgh-2020-003649 (PMC8191610; doi:10.1136/bmjgh-2020-003649)
Supplement: Supplementary data [file bmjgh-2020-003649supp008.pdf]

Supplementary Table 5: Separate interrupted time series analysis for intervention outcome data, without imputation (secondary analysis)

	**Public facilities**							
	**Normal delivery**		**Caesarean section**		**ANC**		**PNC**	
	Estimate (95% CI)	p-value	Estimate (95% CI)	p-value	Estimate (95% CI)	p-value	Estimate (95% CI)	p-value
**Slope change pre-policy**	**1.011(1.001-1.021)**	**0.025**	**1.027(1.018-1.037)**	**0.000**	1.006(0.997-1.014)	0.173	0.997(0.986-1.009)	0.669
**Effect of free maternity policy**								
Level change	**1.285(1.191-1.387)**	**0.000**	**1.345(1.249-1.448)**	**0.000**	**1.091(1.021-1.166)**	**0.010**	0.918(0.837-1.006)	0.068
Trend change	0.997(0.987-1.006)	0.481	**0.981(0.972-0.990)**	**0.000**	0.998(0.990-1.007)	0.723	1.008(0.996-1.020)	0.177
**Effect of Linda Mama policy**								
Level change	1.220(0.931-1.597)	0.149	1.030(0.797-1.332)	0.819	1.272(0.900-1.797)	0.173	0.711(0.441-1.147)	0.162
Trend change	0.995(0.985-1.006)	0.383	**0.984(0.975-0.994)**	**0.002**	0.997(0.987-1.007)	0.575	**1.014(1.000-1.028)**	**0.044**
	**Private and faith-based facilities**							
**Slope change pre-policy**	**1.020(1.003-1.037)**	**0.022**	1.012(0.997-1.026)	0.123	1.013(0.993-1.034)	0.193	**1.037(1.003-1.072)**	**0.030**
**Effect of free maternity policy**								
Level change	**1.215(1.057-1.396)**	**0.006**	1.082(0.959-1.220)	0.200	0.959(0.817-1.125)	0.607	1.250(0.961-1.626)	0.096
Trend change	**0.976(0.959-0.993)**	**0.005**	0.994(0.980-1.009)	0.443	0.991(0.972-1.012)	0.401	**0.966(0.935-0.999)**	**0.043**
**Effect of Linda Mama policy**								
Level change	**0.642(0.424-0.974)**	**0.037**	0.708(0.501-1.001)	0.051	0.825(0.357-1.907)	0.653	0.787(0.198-3.126)	0.734
Trend change	0,995(0.977-1.013)	0.570	1.006(0.990-1.021)	0.476	0.997(0.974-1.021)	0.827	0.979(0.942-1.018)	0.290
